# Bovine papular stomatitis virus as a vaccine vector for cattle

**DOI:** 10.1099/jgv.0.001914

**Published:** 2023-11-17

**Authors:** Gustavo Delhon, Sushil Khatiwada, David Doub, Seth Harris, Sabal Chaulagain, Mostafa El-Gaffary, Daniel L. Rock

**Affiliations:** ^1^​ School of Veterinary Medicine & Biomedical Sciences, University of Nebraska, Lincoln, Nebraska, USA; ^2^​ Department of Pathobiology, College of Veterinary Medicine, University of Illinois, Urbana, Illinois, USA; ^‡^​ Present address: Boehringer Ingelheim Animal Health, Ames, IA, USA; ^§^​ Present address: Department of Molecular Microbiology and Immunology, Bloomberg School of Public Health, Johns Hopkins University, Baltimore, MD, USA; ^#^​ Present address: Department of Veterinary Clinical Pathology, College of Veterinary Medicine, Cairo University, Giza, Egypt

**Keywords:** bovine herpesvirus 1, bovine papular stomatitis virus, challenge, immunization, virus vector

## Abstract

Virus vectored vaccines are not available commercially for cattle even though compelling potential applications exist. Bovine papular stomatitis virus (BPSV), a highly prevalent parapoxvirus, causes self-limited oral lesions in cattle. Ability of virus to accommodate large amounts of foreign DNA, induce low level of antiviral immunity, and circulate and likely persist in cattle populations, make BPSV an attractive candidate viral vector. Here, recombinant BPSV were constructed expressing either Bovine herpesvirus 1 (BoHV-1) glycoprotein gD (BPSV^gD^), or gD and gB (BPSV^gD/gB^). Immunization of BPSV serologically-positive calves with BPSV^gD^ or BPSV^gD/gB^ induced BoHV-1 neutralization antibodies and provided protection for three of four animals following a high dose BoHV-1 challenge at day 70 pi. Results indicate BPSV suitability as a candidate virus vector for cattle vaccines.

Bovine papular stomatitis virus (BPSV), a member of the *Parapoxvirus* (PPV) genus of *Poxviridae*, is a largely neglected virus that causes bovine papular stomatitis (BPS), a highly prevalent mild localized disease of cattle characterized by the presence of erosive papules in the oral cavity, muzzle and, less frequently, udder [1–5]. BPS occurs worldwide, with highest incidence in milking herds and animals less than 1 year-old. While the type PPV orf virus (ORFV) infects sheep and goats and to a lesser extent other ruminants, BPSV has only been detected in cattle, suggesting strong species adaptation. BPSV infection biology and transmission are poorly understood.

Clinical manifestations of BPS appear to be relatively uncommon, and in most cases the infection is either subclinical or very mild, infrequently compromising performance or feeding, or inducing hypersalivation. Unless lesions develop in the integumentary side of lips and/or the muzzle, diagnosis is often overlooked. Transmission occurs by direct contact with lesions or contaminated saliva from infected animals [4]. Similar to orf in sheep and goats, reinfection with BPSV is commonly observed, suggesting that natural virus infection does not confer significant protection against reinfection [1, 6]. The immune response to BPSV is reported to be of short duration, with no significant levels of neutralizing antibodies being elicited by infection [5]. Interestingly, a single report describes protection after challenge of calves with heterologous strains between 24 to 39 days post-infection (pi) [5]. Here, challenge inoculation was performed at the same sites of the primary inoculation, suggesting local antiviral immunity might be responsible [4, 5].

Although available information is limited, BPSV likely establishes some type of persistent infection [7]. Recurrent disease of 15, 33 and 53 % in three different groups of cattle examined periodically have been reported for a period of several years, suggesting that endogenous virus is involved [6]. In a survey in the US, 68 % of oral swabs (*n*=45) collected from healthy calves in a livestock market were positive for BPSV DNA, indicating that subclinically infected cattle are common and a significant reservoir for BPSV transmission in cattle populations [8].

The genetic, biological, and epidemiological similarities between BPSV and ORFV, and current knowledge of ORFV-based vectors make BPSV a potential vaccine vector candidate for cattle. First, it shares all the positive attributes of a poxviral vector, including the ability to accommodate sizeable amounts of foreign DNA. Second, BPSV is highly adapted to cattle in which it establishes subclinical and likely some degree of persistent infection. Viral persistence and subclinical viral replication might be a useful vector attribute as it would promote recall immune responses in the host through repeated antigen presentation over time. Third, the naturally inefficient immune response against BPSV itself may reduce or even abolish anti-vector responses that decrease the performance and utility of many viral vectors, including non-PPV poxvirus vectors [9]. Fourth, the marked tropism of BPSV for mucosal/tegumental surface epithelia makes BPSV-based vectors attractive for specific stimulation of mucosal immunity, which plays critical roles in defence against respiratory pathogens. Fifth, current knowledge on orthologous viral genes affecting virulence, host range and immunomodulatory functions in the related ORFV, allows for rational engineering of attenuated BPSV-based vectors potentially with unique capabilities. Sixth, features above and the ubiquitous nature of BPSV create opportunities for cattle-restricted ‘transmission immunization’ approaches, where a vaccine virus would be transmitted within a population for extended periods of time obviating or reducing the need for additional vaccinations. Finally, a BPSV vectored vaccine would be fully Differentiate Infected from Vaccinated Animal (DIVA) compatible. Notably, viral vectored vaccines are not available commercially for cattle even though compelling potential applications exist, for example improved multivalent vaccines for use against bovine respiratory disease (BRD).

Bovine herpesvirus 1 (BoHV-1) causes various diseases in cattle, including infectious bovine rhinotracheitis (IBR), vulvovaginitis, balanoposthitis, conjunctivitis, and abortion [10]. By targeting the respiratory tract, BoHV-1 plays a leading role in multifactorial BRD, the most costly disease of beef cattle in North America. Both inactivated and attenuated live vaccines are used to control BoHV-1 worldwide. In general, current vaccines are efficient in preventing clinical signs, and some reduce viral spread; however, they cannot prevent infection, including latent infection. To evaluate BPSV vector feasibility, we selected BoHV-1 for proof of principle studies as BoHV-1 infection presents a tractable respiratory disease model for vector evaluation, and protective viral antigens and host responses are established and benchmarked [11]. Here, we report clinical and immune outcomes following vaccination and subsequent BoHV-1 challenge of cattle.

## Construction and characterization of BPSV recombinants containing BoHV-1 gD and gB

**Fig. 1.**
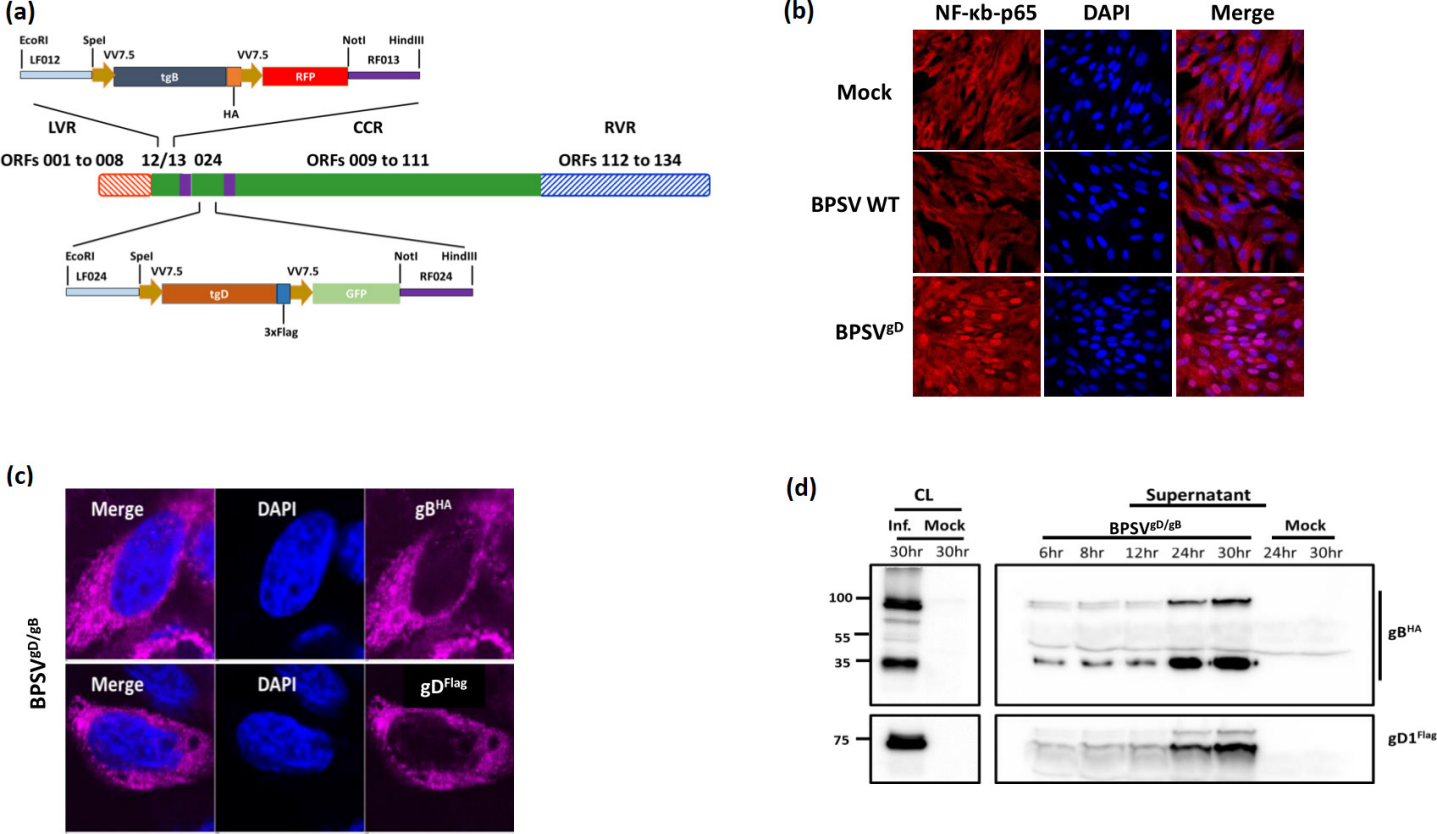
(a) BPSV recombinant viruses obtained by homologous recombination between BPSV strain C5 and recombination cassettes [16]. Top and bottom, structure of synthesized cassettes (GenScript, Piscataway, NJ) used to obtain BPSV^gD/gB^ and BPSV^gD^, respectively. tgB and tgD, HA-tagged truncated BoHV-1 gB and 3×Flag-tagged truncated gD, respectively; VV7.5, early promoter derived from the vaccinia virus 7.5K protein gene; RFP and GFP, red and green fluorescent protein genes, respectively; LF012 and RF013, left and right flanking regions (~500 bp) targeting BPSV012/013 intergenic region; LF024 and RF024, left and right flanking regions (~500 bp) targeting BPSV024 locus. Centre, schematic of BPSV genome, LVR, Left variable region, CCR, Central conserved region, RVR, Right variable region. BPSV^gD^ was obtained first and used as parental virus to obtain BPSV^gD/gB^. Infection/ transfection of OFTu cells and recombinant virus selection and purification was performed as previously described [17]. Integrity and fidelity of inserts were verified by PCR and DNA sequencing, respectively.** (b)** OFTu cells were mock infected or infected with wild-type BPSV C5 (BPSV WT) or BPSV^gD^ (MOI=10). Cells were fixed at 1 h p.i. and incubated with antibody against NF-κB-p65 (no. 8242; Cell Signalling). Cells were then stained with Alexa Fluor 594-labelled secondary antibody and DAPI, and examined by confocal microscopy. Red, NF-κB-p65; blue, DAPI. Approximately 45 % nuclear translocation of NF-κB-p65 was observed in BPSV^gD^-infected cells. (**c)** Subcellular localization of BoHV-1 gB and gD in OFTu cells mock infected or infected with BPSV^gD/gB^ (MOI=3). Cells were fixed at 24hpi, incubated with anti-HA or Flag monoclonal antibody, stained with Alexa Fluor 647-labelled secondary antibody and DAPI, and examined by confocal microscopy [29]. (**d)** Expression of gB^HA^ and gD^Flag^ in OFTu cells mock infected or infected with BPSV^gD/gB^ (MOI=10) for 6, 8, 12, 24 and 30 hpi. Whole cell protein extracts at 30hpi (50 µg) and clarified supernatants from the infected cultures at times indicated were resolved by SDS-PAGE, blotted and transferred to nitrocellulose membranes and probed with antibodies against HA and Flag. Blots were then incubated with appropriate HRP-labelled secondary antibodies and developed using chemiluminescent reagents as described [29].

BoHV-1 major envelope glycoproteins gB, gD, and gC are the primary inducers and targets of neutralizing antibodies, and responses are boosted after reactivation from latency [12, 13]. Subunit vaccines containing the major BoHV-1 glycoproteins have proven effective [14, 15]. A recombinant BPSV C5 strain containing a C-terminal 3×Flag-tagged BoHV-1gD gene lacking transmembrane domain sequences was obtained by homologous recombination in primary ovine fetal turbinate (OFTu) cells, (Fig. 1a, bottom) [16]. In the resulting virus, BPSV^gD^, the gD gene replaces BPSV024, the homolog of ORFV024 (65 % amino acid identity), a nuclear factor κB (NF-κB) inhibitor that prevents NF-κB p65 nuclear translocation during virus infection [17]. To assess whether deletion of BPSV024 affects NF-κB-p65 nuclear translocation, OFTu cells were infected with wild-type BPSV, BPSV^gD^, or mock infected, and NF-κB-p65 localization was examined by immunofluorescence. Infection with BPSV^gD^ but not wild-type BPSV led to rapid nuclear translocation of NF-κB-p65 at 1 h p.i. (Fig. 1b), indicating that BPSV024, like ORFV024, is a functional NF-κB inhibitor.

Using a similar approach and BPSV^gD^ as parental virus, a C-terminal HA-tagged BoHV-1gB gene lacking transmembrane domain sequences was inserted in the BPSV012-013 intergenic region (Fig. 1a, top). The resulting virus BPSV^gD/gB^ as well as BPSV^gD^ exhibited wild-type BPSV replication kinetics in OFTu cell cultures indicating that genetic changes did not impact ability of viruses to replicate *in vitro* (data not shown).

To evaluate BoHV-1gD and gB expression during virus infection, OFTu cells were mock infected or infected with BPSV^gD/gB^ (MOI, 10) and examined by immunofluorescence (IF) with anti-Flag or anti-HA antibodies. gD and gB proteins were observed in the cytoplasm of the infected cells (Fig. 1c). OFTu cells were mock infected or infected with BPSV^gD/gB^ (MOI, 10) and cell lysates and clarified supernatants were examined by Western blot. Protein bands corresponding to gD^Flag^ (approximately 75 kDa) and gB^HA^ (approximately 100, 75 and 35 kDa bands) were observed in both cell lysates and clarified supernatants (Fig. 1d) [18, 19]. Together, BPSV^gD/gB^ efficiently expresses BoHV-1 gD and gB.

## Evaluation of BPSVg^D^ and BPSVg^D/^g^B^ in cattle

**Fig. 2.**
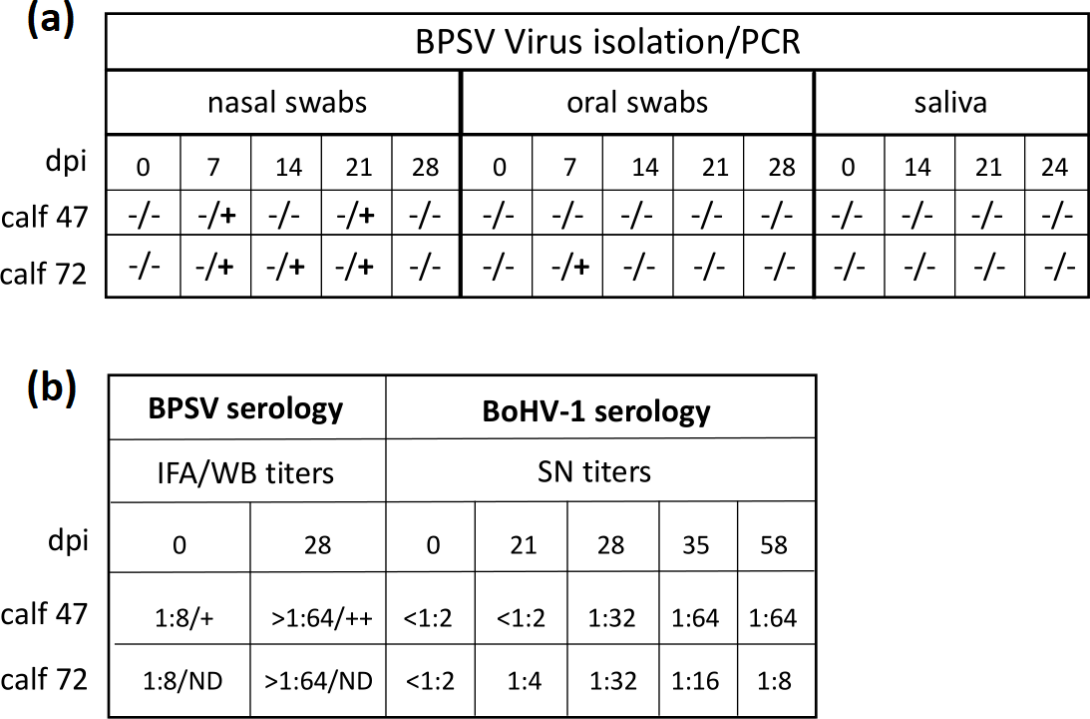
Animal inoculation, Experiment one. Two 4–5 month-old calves (#47 and #72) were inoculated intranasally (0.5 ml/nostril), IM (1 ml/side in the neck) and orally (ID in tongue [two sites] and lower lip [two sites], 100 µl each) with BPSV^gD^ (total viral dose 3.4×10^7^ TCID_50_/calf). Calves were boosted at day 21 pi by the IM route only. (**a) **Nasal and oral swabs and saliva samples were tested at the indicated days post-infection (dpi) for presence (+) of virus (virus isolation in OFTu cells; samples showing no CPE after five blind passages were considered negative) and viral DNA (DNA was extracted with DNA Blood Mini Kit, Qiagen, and PCR run with primers BPSV019FW:CGACTTGTTCACGCGGAGTA and BPSV019RV: CCCCAACAAGTTCGACGCTA) using Phusion Hot Start Flex 2X Master Mix (NEB, Ipswich, MA) following the manufacturer’s instructions. (**b)** Serum samples were tested for antibodies against BPSV by IFA and Western blot [29] and for BoHV-1 seroneutralization (SN) at the indicated times pi. BoHV-1 SN was performed following the Nebraska Veterinary Diagnostic Centre protocol as described in Fig. 3b legend.

*Experiment one*. To evaluate the infection potential and immunogenicity of BPSV^gD^, two 4–5 month-old calves (#47 and #72) serologically positive for BPSV by indirect fluorescence assay (IFA) (Fig. 2b) and negative for BoHV-1 (seroneutralization, SN), were inoculated intranasally (0.5 ml/nostril), IM (1 ml/side in the neck) and orally (intradermal in two sites in the tongue and two sites in the oral side of the lower lip, 100 µl each) with BPSV^gD^ (total virus dose/calf 3.4×10^7^ TCID_50_), and boosted at day 21 pi by the IM route only.

No BPSV lesions were observed at inoculation sites, including the four sites in the oral cavity, indicating BPSV^gD^ is attenuated in the natural host. Infectious virus was not recovered from nasal and oral fluids during 1 month after virus inoculation. However, BPSV DNA sequences were detected by PCR in nasal fluids from both calves between day 7 and 21 post-infection (pi), and from oral fluids on day 7 pi in one calf (Fig. 2a). These results suggest low levels of BPSV replication occurred in inoculated animals, likely providing a source of virus for subsequent transmission. BPSV-specific serum antibody litres were increased from 1 : 8 on day 0 to >1 : 64 on day 28pi in both calves as shown by IFA and Western blot (calf 47) (Fig. 2b). Importantly, BoHV-1 neutralization antibody litres were detected first on day 21 pi in calf 72 (titre 1 : 4) and day 28 in calf 47 (titre 1 : 32) (Fig. 2b). After BPSV^gD^ boost, litres in calf 47 increased to 1 : 64 by day 35 pi and remained unchanged at day 58 pi, while titre in calf 72 peaked on day 28 pi and then declined. This experiment showed that BPSV^gD^ elicited a seroneutralizing antibody response to BoHV-1gD following immunization of BPSV serologically positive animals.

**Fig. 3.**
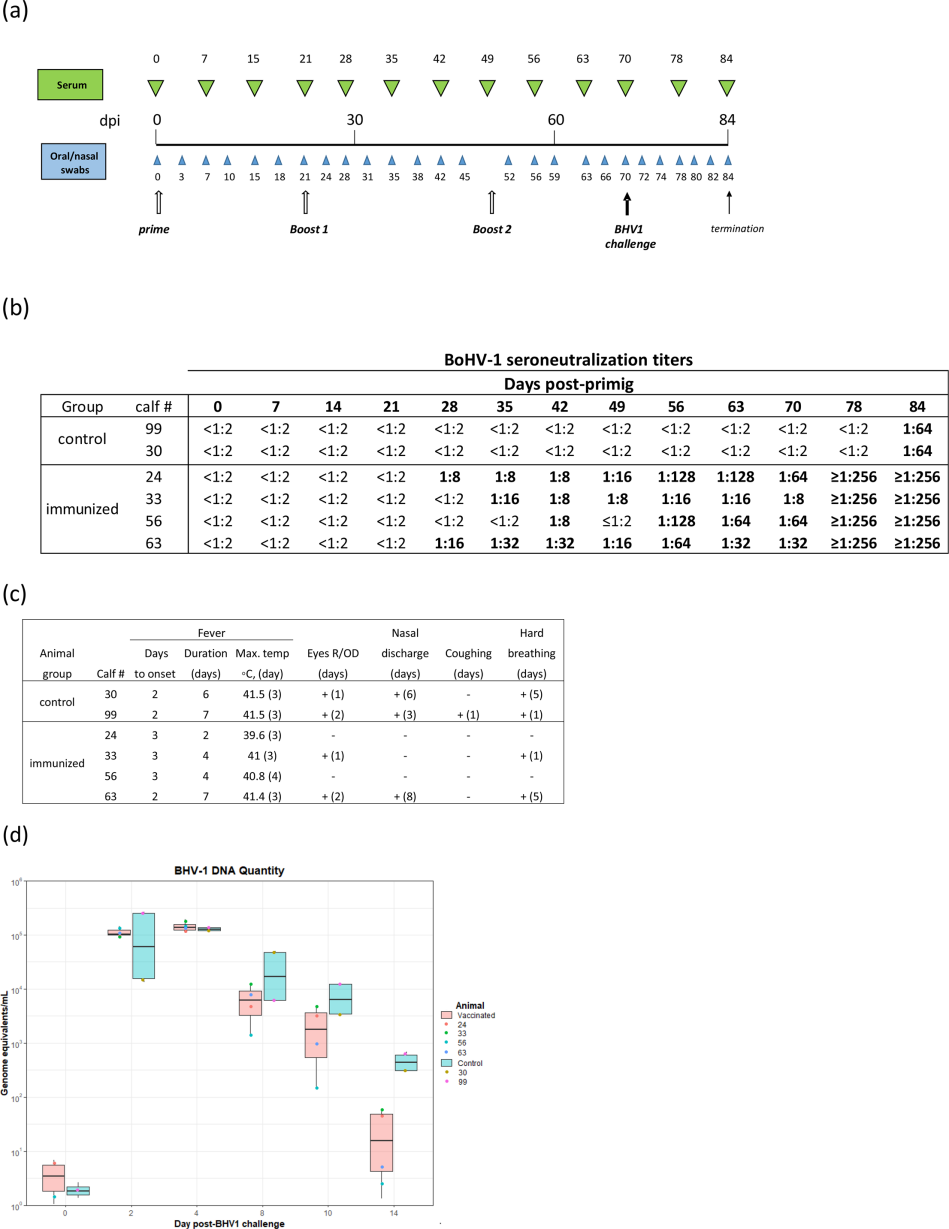
Animal inoculation, Experiment two. (**a)** Experimental design. Six 6-month-old calves were either primed and boosted with BPSV^gD/gB^ (calves 24, 33, 56 and 63) as indicated in the text, or injected with MEM (calves 30 and 99) at the days post-infection (dpi) indicated by the open arrows. Serum samples were collected at times indicated on the top (green arrowheads). Oral and nasal swabs were collected at time points indicated in the bottom (blue arrowheads). All calves were intranasally challenged with BoHV-1 on day 70 post-infection (pi). (**b)** BoHV-1 seroneutralization litres. Serum samples were heat inactivated, diluted 1 : 2 in medium, added by triplicate to 96-well plate columns and mixed with 300 TCID_50_/50 µl BoHV-1 Cooper strain. After 1 h incubation at 37°C, 5 % CO2, BT cells were added, plates incubated and CPE examined. The antibody titre is reported as the highest two-fold dilution of the serum that prevents infectivity of the virus in cell culture; <1 : 2, neutralization not observed. (**c)** BoHV-1 challenge, clinical data. Animals were examined daily between days 0 and 14 post-viral challenge for rectal temperature, and occurrence of nasal and ocular discharges, coughing, and hard breathing [30]. Eyes R/OC, redness/ocular discharge. (**d)** BoHV-1 shedding. Quantitation of BoHV-1 DNA in nasal swabs collected at days 0, 2, 4, 8, 10, and 14 post-challenge was performed using TaqMan qPCR withBoHV-1 primers 1-GAC GAG CTG GGA CTG ATT ATG and 2-GGC AGC GAA ACC ATG AAA TC, and probe /56-FAM/AC GGC ACG G/ZEN/T CGC CTA TAC A/3IABkFQ/. Points represent the means of all three technical replicates per animal.

*Experiment two*. To further examine BPSV as a potential vaccine vector in cattle, recombinant BPSV carrying BoHV-1 gD and gB, BPSV^gD/gB^, was constructed and inoculated into calves. Six 6-month-old BoHV-1- and BVD-negative male calves (SN and IHC tests, respectively) were sorted into two rooms. BPSV preinoculation IFA antibody litres of 1 : 4 to 1 : 8 were detected in the animals. Calves #24, 33, 56 and 63 housed in one room were primed with BPSV^gD/gB^ on day 0 and boosted on days 21 and 49 post-priming. Control calves #30 and 99 housed in a different room received minimal essential medium (MEM) at the same time points. Vector inoculation (virus titre, 10^7^ TCID_50_ ml^−1^) was performed intranasally via nebulization (1 ml/nostril) and IM in the neck (1 ml/side; total virus per calf ~4 x 10^7^ TCID_50_). In addition, the oral mucosa of the upper lip and the muzzle surface were pricked at several sites with a 31G gauge needle embedded in inoculum [5]. Serum samples and nasal and oral swabs were collected at the indicated days post-infection (dpi) (Fig. 3a) and clinical monitoring was performed every 2 days. As in experiment one, clinical signs of BPS were not observed during the immunization period. BoHV-1 neutralizing antibodies were observed 1 week after the first boost in immunized calves #24 and 63 and in the remaining immunized calves 1 to 2 weeks later. At the time of challenge with BoHV-1, serum neutralizing titers in immunized calves ranged from 1 : 8 to 1 : 64.

On experimental day 70, all six calves were challenged intranasally via nebulization with a high dose of BoHV-1 Cooper strain (2×10^8.5^ TCID_50_/animal), and monitored daily for clinical signs. Blood samples and oral and nasal swabs were collected at days indicated in Fig. 3(a). All calves presented fever starting on post-challenge (pc) days 2 or 3, and lasting an average of 6.5 days in control animals and 4.2 days in immunized animals (Fig. 3c). The two control calves, #30 and #99, presented ocular and nasal discharges and laboured breathing, and one of them (#99), coughing. In the immunized group, the two calves with the highest neutralizing antibody litres, #24 and #56, exhibited no clinical signs other than low level and short duration fever, while calf #33 showed additional clinical signs for only a single day (Fig. 3c). Calf #63 exhibited clinical signs indistinguishable from control calves (Fig. 3c). BoHV-1 DNA in nasal swabs declined gradually post-challenge but values did not differ statistically between groups; however, at later time points, amounts trended lower in vaccinated animals (Fig. 3d).

The results described here indicate that immunization of calves with BPSV vectors containing heterologous BoHV-1 proteins gB and gD, induced neutralizing antibodies and provided protection from BoHV-1 challenge infection. Notably and of likely consequence for potential future applications, the BPSV vector was effective in inducing immune responses in BPSV serologically-positive animals. This result is consistent with the known high reinfection potential of BPSV and its high prevalence and transmissibility in bovine populations [6, 8]. Thus, prior BPSV infection does not pose a significant obstacle for a BPSV-based vector.

No adverse effects were observed in BPSV^gD^ and BPSV^gD/gB^ immunized animals. Deletion of BPSV024, a functional NF-κB inhibitor (Fig. 1d) from the viral vector may have contributed to the observed attenuation; however, its actual role remains to be determined, as clinical manifestations of BPS infection appear to be relatively uncommon in cattle even though infection is widespread in bovine populations [8].

Notably, BoHV-1 neutralizing antibody titers obtained following inoculation with BPSV^gD^ and BPSV^gD/gB^ were comparable or higher than those observed following vaccination with a widely used commercial BoHV-1 LAV vaccine, VistaOnce SQ (Merck Animal Health) [20]. And, protection from overt clinical disease was observed in three of four vaccinated animals following challenge infection. Notably, the challenge dose used here (2×10^8.5^ TCID_50_/animal) was markedly higher than doses routinely used in BoHV-1 animal challenge experiments (approximately 10^7.0^ TCID_50_/animal) [21–23]. Interestingly, the protection observed in these animals appeared to be independent of early virus replication and nasal shedding (Fig. 3d). This result suggests that, as shown for immunization with individual BoHV-1 glycoproteins, the BPSV vector-induced immunity observed may not be dependent on early reduction of BoHV-1 virus replication in the upper respiratory tract [24]; however, the actual significance of this single observation and the consistency of this finding remain to be determined.

While the results shown here are promising, there are numerous additional opportunities to improve aspects of BPSV vector immunogenicity. For example, optimizing the route for vector administration may be of benefit; currently BPSV target sites in the oral mucosa/upper respiratory tract are poorly described. Adjustments here may lead to optimal vaccine delivery, enhancing host responses. Additionally, the BPSV genome easily can be engineered to accommodate additional BoHV-1 protective antigens. Further, the emerging understanding of the genetic basis of PPV virulence and immunomodulation should permit construction of vectors with unique antigenic and immunomodulatory properties. For example, BPSV encodes homologs of ORFV immunomodulators ORFV024, ORFV073, ORFV113, ORFV119, and ORFV121 [17, 25–29]. Conceivably, these BPSV homologs can be targeted for modulation of immunogenicity of vectored antigens, further facilitating induction of robust protective responses. Such strategies will require additional studies on BPSV immunomodulators in the natural host.

Based on results shown here, BPSV shows promise as a vector for use in cattle especially where a multivalent vaccine is required, such as with BRD. Although important aspects of BPSV infection/transmission biology in nature remain poorly defined, BPSV vectors may offer unprecedented utility; an ability to establish persistent/latent infections, high host reinfection potential and highly efficient transmission characteristics may translate into ‘self-boosting vaccines’ performing at both the individual and population level. A ‘self-boosting vaccine’ would enhance the magnitude and duration of host immune responses to target antigens; this would be of particular advantage where longer duration immunity is desirable, such as in range production systems and dairy animals. Perhaps more importantly, these vectors may have potential as ‘perpetual vaccines’ where a single vaccine dose will control target diseases within an animal population in perpetuity via ‘transmission immunization’, thus delivering the benefits of vaccination at almost no cost. This strategy may reduce or remove the need for repeated vaccinations. If successful, ‘perpetual vaccines’ may address intractable animal disease control challenges associated with wildlife and livestock in the developing world.
